# Symbiotic Performance of Diverse *Frankia* Strains on Salt-Stressed *Casuarina glauca* and *Casuarina equisetifolia* Plants

**DOI:** 10.3389/fpls.2016.01331

**Published:** 2016-08-31

**Authors:** Mariama Ngom, Krystelle Gray, Nathalie Diagne, Rediet Oshone, Joel Fardoux, Hassen Gherbi, Valérie Hocher, Sergio Svistoonoff, Laurent Laplaze, Louis S. Tisa, Mame O. Sy, Antony Champion

**Affiliations:** ^1^Laboratoire Mixte International Adaptation des Plantes et microorganismes associés aux Stress Environnementaux, Centre de Recherche de Bel-AirDakar, Sénégal; ^2^Laboratoire Campus de Biotechnologies Végétales, Département de Biologie Végétale, Faculté des Sciences et Techniques, Université Cheikh Anta DiopDakar, Sénégal; ^3^Laboratoire Commun de Microbiologie Institut de Recherche pour le Développement/Institut Sénégalais de Recherches Agricoles/Université Cheikh Anta Diop, Centre de Recherche de Bel-AirDakar, Sénégal; ^4^UMR DIADE, Institut de Recherche pour le DéveloppementMontpellier, France; ^5^Centre National de Recherches Agronomiques, Institut Sénégalais de Recherches AgricolesBambey, Sénégal; ^6^Department of Molecular, Cellular, and Biomedical Sciences, University of New HampshireDurham, NH, USA; ^7^Laboratoire des Symbioses Tropicales et Méditerranéennes, Institut de Recherche pour le Développement/INRA/CIRAD/Université Montpellier/Sup agroMontpellier, France

**Keywords:** salinity, *Frankia*, *Casuarina glauca*, *Casuarina equisetifolia*, root hair deformation, *CgNIN*, *Cg12*, proline

## Abstract

Symbiotic nitrogen-fixing associations between *Casuarina* trees and the actinobacteria *Frankia* are widely used in agroforestry in particular for salinized land reclamation. The aim of this study was to analyze the effects of salinity on the establishment of the actinorhizal symbiosis between *C. glauca* and two contrasting *Frankia* strains (salt sensitive; CcI3 vs. salt tolerant; CeD) and the role of these isolates in the salt tolerance of *C. glauca* and *C. equisetifolia* plants. We show that the number of root nodules decreased with increasing salinity levels in both plants inoculated with CcI3 and CeD. Nodule formation did not occur in seedlings inoculated with CcI3 and CeD, at NaCl concentrations above 100 and 200 mM, respectively. Salinity also affected the early deformation of plant root hairs and reduced their number and size. In addition, expression of symbiotic marker *Cg12* gene, which codes for a subtilase, was reduced at 50 mM NaCl. These data suggest that the reduction of nodulation in *C. glauca* under salt stress is in part due to inhibition of early mechanisms of infection. We also show that prior inoculation of *C. glauca* and *C. equisetifolia* with *Frankia* strains CcI3 and CeD significantly improved plant height, dry biomass, chlorophyll and proline contents at all levels of salinity tested, depending on the *Casuarina-Frankia* association. There was no correlation between *in vitro* salt tolerance of *Frankia* strains and efficiency *in planta* under salt-stressed conditions. Our results strongly indicate that increased N nutrition, photosynthesis potential and proline accumulation are important factors responsible for salt tolerance of nodulated *C. glauca* and *C. equisetifolia*.

## Introduction

Soil salinization is a major problem worldwide. Indeed, high levels of salt in soil limit crop production and increase the loss of arable land. More than 800 million hectares of land worldwide are salt-affected (Munns and Tester, 2008). By the year 2050, 50% of all arable lands could be affected by salinity (Wang et al., 2003). There is therefore a need to design strategies to rehabilitate salinized areas.

Actinorhizal plants belonging to *Casuarinaceae* family such as *Casuarina glauca* and *C. equisetifolia* are able to grow under saline environments (El-Lakany and Luard, 1983; Girgis et al., 1992; Tani and Sasakawa, 2003). They are fast-growing trees, originated from Australia and Pacific islands, widely used in agroforestry systems for several purposes (Diem and Dommergues, 1990). In many tropical and sub-tropical countries, *Casuarina* species play a major role in land reclamation, crop protection and as windbreaks (National Research Council, 1984). In Senegal, a green barrier of *C. equisetifolia* was established on the northern Atlantic fringe between Dakar and Saint-Louis to stabilize sand dunes and protect the vegetable and fruit producing so-called “Niayes” area (Maheut and Dommergues, 1961; Mailly et al., 1994). *C. equisetifolia* is also appreciated for source of poles, firewood and charcoal (Diagne et al., 2013; Potgieter et al., 2014). Thus, this family of plants is of high importance for salinized land reclamation.

*Casuarina* species are pioneer plants, able to colonize poor and degraded lands and increase their fertility (Duhoux and Franche, 2003). Therefore, they promote development of pedogenetic processes leading to the formation of a more suitable microclimate for the installation of other plants species (Moiroud, 1996). This property is mainly due to the tremendous plasticity of their root system allowing them, among other things, to establish a nitrogen-fixing actinorhizal symbiosis with a filamentous soil bacterium called *Frankia*. Nitrogen is one of the main factors limiting plant growth and crop production worldwide, despite being the most abundant element in the atmosphere (80%). Unlike nitrogen-fixing plants, the majority of plant species are unable to directly utilize atmospheric nitrogen and rely on poor nitrogen sources in soils for their nutrition (Santi et al., 2013).

Among *Casuarina* species, *C. glauca* and *C. equisetifolia* display a high salt tolerance (El-Lakany and Luard, 1983). In addition, *C. glauca* is a model tree for basic and fundamental research in actinorhizal symbiosis with the development of many tools including genetic transformation of *C. glauca* and transcriptome analyses (Smouni et al., 2002; Gherbi et al., 2008; Tromas et al., 2012; Svistoonoff et al., 2013, 2014; Diédhiou et al., 2014; Champion et al., 2015). They are therefore good models to study the mechanisms involved in tolerance to salt stress in actinorhizal trees. One important question is how salt stress impacts actinorhizal symbioses establishment. The early steps of the infection process leading to the development of root nodules of *Casuarina* tree starts with the induction of root hair curling by *Frankia*, as early as 24 h after inoculation (Callaham et al., 1979; Perrine-Walker et al., 2011). *Frankia* hyphae proceed to penetrate the host plant through a deformed root hair (Franche et al., 1998) and induce the expression of several plant genes involved in actinorhizal nodule formation and functioning. Among them are the *CgNIN*, encoding a transcriptional factor, expressed at pre-infection stages in root hairs competent for *Frankia* infection (Clavijo et al., 2015) and *Cg12*, encoding a subtilase, whose expression is linked to the infection of root hairs and cortical cells by *Frankia* (Laplaze et al., 2000; Svistoonoff et al., 2003).

*Frankia* is a genus of soil actinobacteria (Normand et al., 2014). These Gram+, aerobic, heterotrophic bacteria are able to fix nitrogen both under free-living conditions and inside symbiotic root nodule (Simonet et al., 1989). The first pure culture of a *Frankia* strain was isolated from *Comptonia peregrina* nodules (Callaham et al., 1978). Since then, several *Frankia* strains have been isolated from different actinorhizal species (Diem et al., 1982, 1983; Gomaa et al., 2008; Gtari et al., 2015). They are grouped into four major clusters (Normand et al., 1996). *Frankia* strains in cluster 1 form nodules either with members of *Betulaceae* and *Myricaceae* (cluster 1a) or *Casuarinaceae* (cluster 1c). Cluster 2 includes *Frankia* strains able to infect the *Coriariaceae, Datiscaceae, Rosaceae*, and *Ceanothus* of the *Rhamnaceae*. *Frankia* strains in cluster 3 form effective nodules with the *Myricaceae, Rhamnaceae, Elaeagnaceae*, and *Gymnostoma* belonging to *Casuarinacea*e. Cluster 4 includes atypical *Frankia* strains, which are non-infective and/or non-nitrogen-fixing. *Casuarina* isolates show contrasting responses in their salt tolerance (Ngom et al., 2016). Indeed, some strains are more tolerant *in vitro* to salt stress than others even though they were isolated from the same host plant (Dawson and Gibson, 1987; Fauzia, 1999; Tani and Sasakawa, 2003; Oshone et al., 2013). Nevertheless, the symbiotic performance under salt stress of diverse *Frankia* strains toward *Casuarinaceae* species remains poorly understood.

In this study we aim to analyze (i) the effects of salinity on the establishment of symbiosis between *C. glauca* and two *Frankia* strains: CcI3 (a salt sensitive strain) vs. CeD (a salt tolerant strain) and (ii) the role of these isolates in salt tolerance in *C. glauca* and *C. equisetifolia*.

## Materials and methods

### Bacterial material and growth conditions

Two contrasting *Frankia* strains were used in this study. CcI3 strain, whose isolation was reported by Zhang et al. (1984) is sensitive to salt stress while CeD, isolated by Diem et al. (1982) is salt tolerant in our cultivation conditions. Both *Frankia* isolates were grown in liquid BAP medium which contained (at final concentration) 1.4 mM CaCl_2_·2H_2_O, 0.2 mM MgSO_4_·7H_2_O, 0.195 mM FeNaEDTA, 5.6 mM KH_2_PO_4_, 3.2 mM K_2_HPO_4_, trace elements (H_3_BO_4_, M_n_Cl_2_·4H_2_O, ZnSO_4_·7H_2_O, CuSO_4_·5H_2_O, Na_2_MO O_4_·2H_2_O, and CoSO_4_·7H_2_O) and vitamins (thiamine-HCL, pyridoxine-HCL, folic acid, Ca panthotenate, nicotinic acid, biotin, and riboflavin) at a final pH of 6.7 (Murry et al., 1984). Sodium propionate (5 mM) and NH_4_Cl (5 mM) were used as carbon and nitrogen sources, respectively. For rapid hyphal growth, this nutrient BAP medium was modified and supplemented with phosphatidyl choline (3.33 g/L) and MES-Tris buffer (0.5 M, pH 6.8; Schwencke, 1991). Cultures were maintained at 28 ± 1°C, in darkness under stirring conditions.

### Plant material, plant transformation growth conditions

*C. glauca* seeds (seed lot 15,934, ref. 086-5929) were collected at the Myall Lakes National Park in Australia and provided by the Australian Tree Seed Centre (ATSC, CSIRO). *C. equisetifolia* seeds (seedlot SN/2011/0014/D) were collected in Louga area in Senegal and provided by the National Tree Seed Program (PRONASEF).

For experiments with non-trangenic plants, *C. glauca* and *C. equisetifolia* seeds were germinated under semi axenic conditions in a plastic tray (53.5 × 27.5 cm) containing a sterile mixture of compost (ref EN 12580) and sandy soil (v/v; 120°C, 60 min). They were watered daily with a quarter-strength Hoagland liquid medium (Hoagland and Arnon, 1950) to promote germination and initial growth of the seedlings.

Genetic transformation of *C. glauca* was performed using an *Agrobacterium tumefaciens* strain containing a Pro*Cg12:GFP* construct (Svistoonoff et al., 2003). Six independent *C. glauca* transgenic lines were generated as described previously (Smouni et al., 2002). For each transgenic line, GFP expression was analyzed. All plants showed the expression pattern described in Svistoonoff et al. (2003). The Pro*Cg12:GFP* line showing the highest expression levels of GFP was clonally propagated as described (Svistoonoff et al., 2010). Similarly for Pro*CgNIN:GFP*, we used the transgenic line previously described (Clavijo et al., 2015) which showed the highest GFP expression.

### Effect of salinity on nodulation of *C. glauca* plants

One month after seed germination, *C. glauca* seedlings were uprooted from the soil, gently washed 5 times with distilled water. Seedlings were individually transferred in hydroponic conditions, into Gibson glass tubes filled with a 50 mL liquid BD medium supplemented with KNO_3_ (5 mM) as nitrogen source, at pH 6.7 (Broughton and Dilworth, 1971). They were incubated in a growth chamber at 28 ± 1°C with 16 h day/8 h night photoperiod and a 74 μmol m^−2^ s^−1^ light intensity. The BD medium was renewed every 2 weeks to avoid nutrient depletion and pH drift. After 1 month, salt stress was applied gradually through the weekly increment of one concentration of NaCl at 0, 50, 100, 200, 300, 400, and 500 mM. When 500 mM NaCl was reached, the plants were placed in nitrogen free-BD medium before being inoculated separately either with CcI3 or CeD *Frankia* strains.

*C. glauca* nodulation was performed as described previously (Ngom et al., 2015). Before inoculation, homogenized cells of CcI3 and CeD were suspended in sterile water with a final absorbance of 0.2, measured at λ = 595 nm for each strain. To establish actinorhizal symbiosis, inoculum of each strain was first brought into contact with the root system for 2 h. Plants were replaced back into Gibson tubes replenished with a 45 mL of nitrogen free-BD medium +5 mL of each bacterial suspension. Nodulation rate or the percentage of nodulated plants (total number of nodulated plants/total number of inoculated plants × 100) and the mean nodule number (average number of nodules per plant) were followed for about 2 months after inoculation. All experiments were repeated twice and 22 plants were used for each salt treatment per experiment.

### Effect of salinity on *C. glauca* root hair deformation

*C. glauca* seedlings were placed in hydroponic culture. Salt stress was applied gradually and plants were inoculated separately with either *Frankia* strain CcI3 or *Frankia* strain CeD, as described above. Two days after inoculation, root hair deformation was evaluated through micrographs of small lateral roots acquired with a Micro Publisher 3.3 RTV digital camera (QImaging) and a BX50F microscope (Olympus). For each treatment, five plants were used and three lateral roots were analyzed per plant. A total of 180 lateral roots and 12,217 root hairs were observed. Root hair deformation intensity was evaluated as described in Clavijo et al. (2015). For each micrograph, root hairs were observed and the following scoring was used: 0, no deformation; 1, straight root hair with tip swelling; 2, only one change in growth direction; 3, more than one change in growth direction but no bifurcation; 4, one or more bifurcations. At least two independent experiments were performed.

### Analysis of *CgNIN* and *Cg12* activation under salinity

Transgenic lines expressing Pro*CgNIN:GFP* or Pro*Cg12:GFP* fusions were propagated and grown hydroponically in BD medium as described previously (Svistoonoff et al., 2010). Two NaCl concentrations (0 and 50 mM) were applied for 7 days. Plants were inoculated either with *Frankia* strain CcI3 or CeD, as described above. For each transgenic line, four plants per treatment were used. Activation of Pro*CgNIN:GFP* was monitored 24, 48, and 72 h after inoculation. Activation of Pro*Cg12:GFP* was observed 3, 7, and 14 days after inoculation and nodule sections were examined for GFP fluorescence. GFP expression was observed using an AZ100 epifluorescence microscope (Nikon) and a GFP filter.

### Effects of prior inoculation with *Frankia* on the salt tolerance of *C. glauca*

*C. glauca* seedlings were cultivated in hydroponic conditions and were nodulated with *Frankia* strains CcI3 or CeD, as described above. A batch of 22 uninoculated plants was used as controls. After inoculation, nodule formation was monitored weekly. Twenty-five days after inoculation, all of the plants were nodulated and treatment with NaCl was initiated. Salt stress (0, 50, 100, 200, 300, 400, and 500 mM NaCl) was applied gradually, as described above, to avoid osmotic shock. Morphological and physiological parameters of growth such as length of aerial parts, shoot and root dry weight, chlorophyll, and proline contents were evaluated as described below. Independent experiments were performed twice with 22 plants each treatment per experiment.

### Effects of prior inoculation with *Frankia* on the salt tolerance of *C. equisetifolia*

One month after seed germination, *C. equisetifolia* seedlings were transplanted into plastic bags containing sterile sandy soil (120°C, 1 h). The experiments were conducted in a nethouse (Bel-Air experimental station, 14°44′N–17°30′W, Dakar, Senegal). Seedlings were watered daily and inoculation was applied 1 month after transplantation. Suspension of crushed nodule was used as inoculum. Nodules (20 g) were collected from *C. glauca* plants grown in hydroponic conditions and inoculated separately with *Frankia* strains CcI3 or CeD. Nodules were surface-sterilized with 5% sodium hypochlorite for 20 min then rinsed 3 times in sterile distilled water as described by Ng (1987). Grounded nodules were resuspended in 500 mL sterile distilled water. A 5 mL suspension was added into each bag according to the *Frankia* strain except for uninoculated plants. A batch of 8 plants was used for each treatment. As for *C. glauca*, the establishment of the symbiosis was monitored before gradually applying salt stress (0, 50, 100, 200, 300, 400, and 500 mM NaCl), as described above. Morphological and physiological parameters of growth such as length of aerial parts, shoot and root dry weight, chlorophyll, and proline contents were evaluated as described below.

### Growth of aerial part and dry weight determination

Length of aerial parts were measured every 2 weeks. Four months after inoculation, plants were harvested. Shoot and root systems were collected, washed in deionized water, surface-wiped with blotting paper, and dried at 70°C for 72 h. The dried biomasses of each samples (*C. glauca n* = 22, *C. equisetifolia n* = 8 per sample) were weighed separately.

### Measurement of chlorophyll content

Chlorophyll content was determined using Arnon's method (1949). Fresh leaves (100 mg) were crushed in 10 mL of acetone at 80%. Samples were incubated overnight at 4°C and centrifuged at 6000 g for 10 min. The absorbance of chlorophyll (a) and (b) was measured using a UV-1800 spectrophotometer (UVisco) at λ = 663 and 645 nm, respectively. Total chlorophyll content (*C. glauca n* = 5, *C. equisetifolia n* = 4 per sample) was calculated according to Arnon (1949).

### Extraction and measurement of proline content

Fresh leaves (100 mg) were crushed in 2 mL of methanol at 40%, and the samples were immerged in a water bath at 85°C for 1 h. After cooling, 1 mL of leaf extract was mixed with 1 mL of ninhydrin at 2.5% and 1 mL of the reaction mixture (48 mL distilled water, 32 mL acetic acid, and 120 mL orthophosphoric acid). A second incubation was done in a water bath at 100°C for 30 min. Samples were cooled on ice, then a 5 mL toluene was added to the mixture. The upper phase was collected after vortexing and dehydrated with anhydrous sodium sulfate. Absorbance of leave samples was measured using a spectrophotometer at λ = 520 nm, as described by Monneveux and Nemmar (1986). Proline contents (*C. glauca n* = 5, *C. equisetifolia n* = 4 per sample) were calculated and determined through a calibration straight graph constructed from a standard range of proline concentrations (Monneveux and Nemmar, 1986).

### Acetylene reduction assay (ARA)

Nitrogen fixation was measured using the acetylene reduction assay described by Hardy et al. (1973). *C. glauca* plants were placed in tightly closed 150 mL jars. In each jar, 10% of the air (15 mL) was removed and replaced with acetylene. Plants were incubated at 28°C for 3 h. From each jar, 1 mL was withdrawn and assayed for ethylene using a gas chromatograph (Agilent 6850, GC System). Nodules were removed from plant roots and dried at 70°C for 72 h. Nitrogenase activity was calculated per nodule dry weight and expressed as nmoles ethylene/nodule (g).

### Statistical analysis

Statistical analyses were performed on dry weight, chlorophyll and proline data. Statistical tests were performed using the XLSTAT 7.2 software. The Student-Newman–Keuls test at *p* < 0.05 was used to evaluate the differences between inoculated and uninoculated plants and between NaCl treatments.

## Results

### Effects of salt and osmotic stresses on the growth of *Frankia* strains CcI3 and CeD

**Figure 1:**
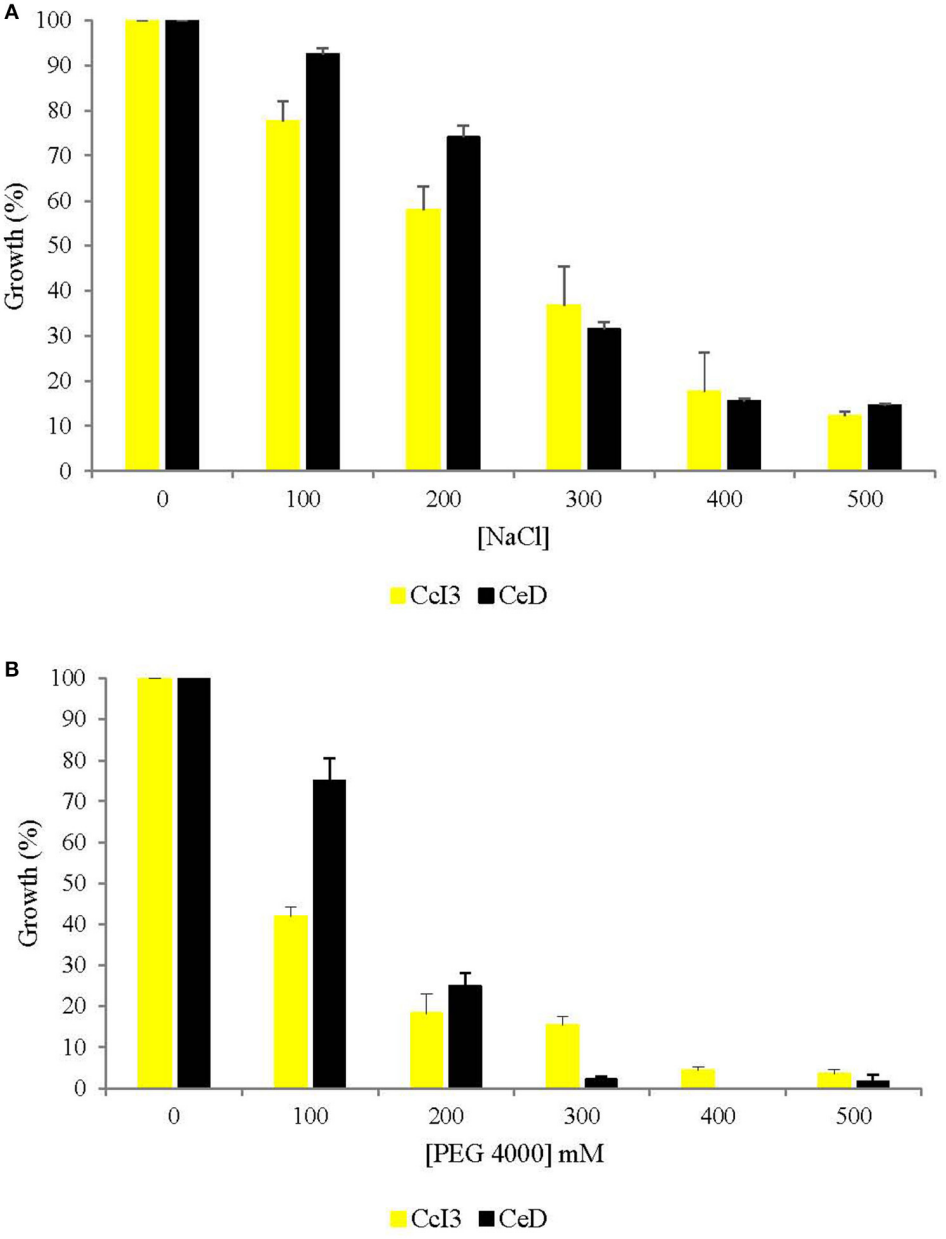
**Effect of salt and osmotic stresses on the growth of ***Frankia*** strains Ccl3 and CeD**. Cultures were grown under several concentrations of NaCl **(A)** and Polyethylene Glycol 4000 **(B)** for 7 days. Growth of each *Frankia* strain was estimated by measuring the turbidity at λ = 595 nm. The growth of *Frankia* in absence of salt and osmotic stresses (100% growth) was compared with those in the presence of NaCl or PEG 4000. Vertical bars indicate the standard error of mean (2 biological and 8 technical replicates). The absence of error bars indicates that the size of the error does not exceed the size of the symbol.

First we analyzed the growth of 8 *Frankia* strains isolated from several *Casuarina* species under saline conditions. All of the strains showed a reduced growth in response to salt treatment (data not shown). Two *Frankia* strains (CcI3 and CeD) were selected on the basis of their different sensitivity to salt and osmotic stresses. As shown in Figure 1, the growth of both *Frankia* isolates was reduced by increasing the NaCl and PEG concentrations in the medium. At 100 and 200 mM NaCl or PEG, the growth of CeD was significantly less impacted than the growth of CcI3 (Figures 1A,B). At high concentrations of NaCl and PEG (300, 400, and 500 mM), no or reduced growth was observed for both strains, with a more pronounced effect in presence of PEG 4000 in the medium. Altogether, our data indicated that CeD is more tolerant to salt and osmotic stresses than CcI3.

### Salinity inhibits *C. glauca* plant nodulation

The impact of different NaCl concentrations on the nodulation of *C. glauca* plants by *Frankia* strains CcI3 and CeD was studied (Table 1). In both plants inoculated with *Frankia* CcI3 and CeD, the control plants (0 mM NaCl) had higher mean nodule number and rate of nodulation than NaCl-treated plants. The number of nodules formed increased over time at different rates with more nodules on seedlings inoculated with *Frankia* strain CeD for 63 days. At 50 mM NaCl, the mean nodule number declined by 66.6 and 60.3% in plants inoculated with CcI3 and CeD, respectively, compared to control plants. Nodule formation did not occur in seedlings inoculated with strain CcI3 at NaCl concentrations above 100 mM, whereas some plants inoculated with strain CeD were still forming nodules at 200 mM NaCl after 49 days of inoculation. Mean nodule number and nodulation rate of seedlings were reduced by increasing the salinity level.

**Table 1:** **Effects of several concentrations of NaCl on the nodulation of ***C. glauca*** plants inoculated with ***Frankia*** strains CcI3 and CeD**.

***Frankia* strains**	**NaCl treatments (mM)**		**Nodulation kinetic (Days after inoculation)**							
			**14**	**21**	**28**	**35**	**42**	**49**	**56**	**63**
CcI3	0	Nodulation rate (%)	72.7	95.5	100	100	100	100	100	100
		Mean nodule number	4.4 ± 1.1	9.9 ± 1.7	27.9 ± 3.4	36.1 ± 4.2	40.4 ± 4.4	42.2 ± 4.1	44.1 ± 4.2	45.8 ± 4.2
	50	Nodulation rate (%)	36.4	59.1	86.4	86.4	90.9	90.9	90.9	95.5
		Mean nodule number	0.8 ± 0.3	3 ± 0.8	7.4 ± 1.4	9.6 ± 1.9	10.2 ± 2	11.7 ± 1.9	13.4 ± 2.2	15.3 ± 2.3
	100	Nodulation rate (%)	4.5	18.2	40.9	40.9	40.9	40.9	40.9	40.9
		Mean nodule number	0.05 ± 0.05	0.4 ± 0.2	2.4 ± 0.9	3.6 ± 1.3	3.7 ± 1.4	4.2 ± 1.6	4.5 ± 1.7	4.9 ± 1.8
	≥200	Nodulation rate (%)	0	0	0	0	0	0	0	0
		Mean nodule number	0	0	0	0	0	0	0	0
CeD	0	Nodulation rate (%)	50	90.9	100	100	100	100	100	100
		Mean nodule number	2.3 ± 0.7	11 ± 2	28 ± 3.3	37.1 ± 3.3	38.8 ± 3.3	44.8 ± 3.4	48.8 ± 3.5	50.3 ± 3.5
	50	Nodulation rate (%)	18.2	50	68.2	68.2	68.2	81.8	81.8	81.8
		Mean nodule number	0.5 ± 0.3	2.7 ± 0.8	7.1 ± 1.5	12.2 ± 2.5	13.5 ± 2.6	16.7 ± 2.8	18 ± 3	20 ± 3.2
	100	Nodulation rate (%)	4.5	9.1	22.7	40.9	40.9	59.1	63.6	68.2
		Mean nodule number	0.05 ± 0.05	0.1 ± 0.1	3 ± 1.2	4.6 ± 1.6	4.7 ± 1.6	7.9 ± 2.3	9.1 ± 2.4	9.7 ± 2.5
	200	Nodulation rate (%)	0	0	0	0	0	9.1	9.1	22.7
		Mean nodule number	0	0	0	0	0	0.5 ± 0.3	0.6 ± 0.4	0.8 ± 0.5
	≥300	Nodulation rate (%)	0	0	0	0	0	0	0	0
		Mean nodule number	0	0	0	0	0	0	0	0

*Salt stress was first gradually applied then plants were inoculated separately with each Frankia strain. Nodulation rate is the percentage of plant nodulated and mean nodule number is the average number of nodule per plant. Values represent the mean ± standard deviation of plants used in each treatment (n = 22)*.

### Salinity severely affects root hair deformation response to *Frankia* inoculation

**Figure 2:**
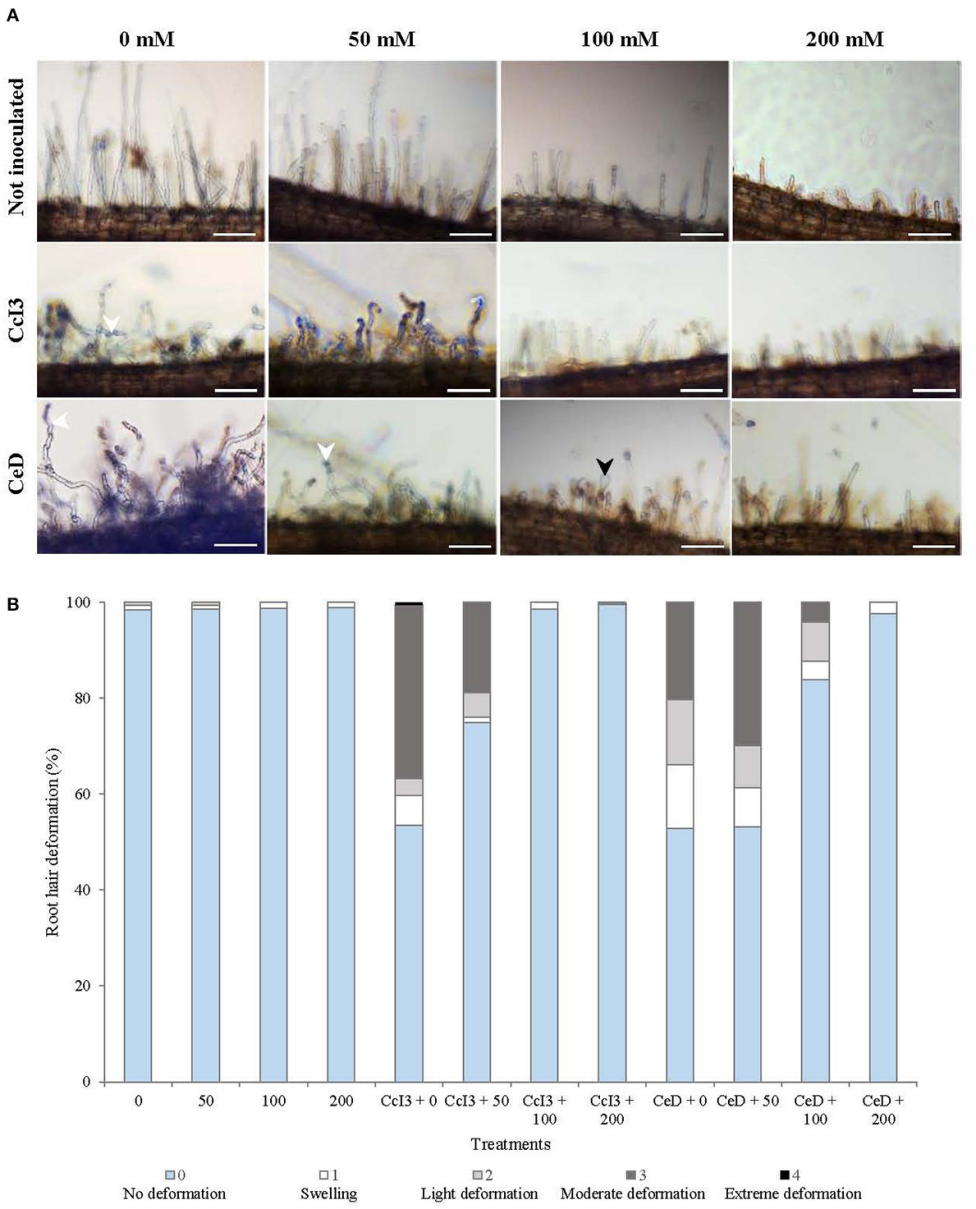
**Effect of various levels of NaCl on ***C. glauca*** root hair deformation**. **(A)** Root hairs under salt-stressed conditions from uninoculated and inoculated *C. glauca* plants, observed 2 days after inoculation. White arrows indicate moderate deformation and black arrows swelling root hairs (Bars, 100 μm). **(B)** Quantification of root hair deformation showing the proportion of deformed root hairs in short lateral roots 2 days after inoculation with *Frankia* strains Ccl3 and CeD. For each treatment, 5 plants were used and 3 lateral roots were observed per plant.

Because salinity inhibited nodulation, the effects of salt stress during the early stages of the actinorhizal symbiosis establishment was investigated. Root hair deformation responses in *C. glauca* plants treated with several concentrations of NaCl and inoculated with *Frankia* strains CcI3 and CeD was first analyzed. Regardless of the presence of *Frankia*, salt treatment reduced the number and size of root hairs in both uninoculated and inoculated *C. glauca* plants (Figure 2A). In *C. glauca* plants inoculated with *Frankia* strains CcI3 and CeD, extensive root hair deformation was detected 2 days after inoculation, in small lateral roots of no salt-treated plants. Increased salinity reduced the amount of deformation in seedlings inoculated by both strains with a more pronounced effect for plants inoculated with CcI3 (Figure 2B). Deformation was particularly low at 200 mM NaCl, which also showed previously the smallest number of nodules in plants inoculated with CeD and no nodule development in plants inoculated with CcI3. No or few deformation were observed on uninoculated plants treated with various levels of NaCl.

### Effects of salinity on *CgNIN* and *Cg12* expression

To further investigate the effects of salinity during the early stages of the establishment of symbiosis, we studied the impact of salt stress on the expression of two early symbiotic marker genes: *CgNIN* (Clavijo et al., 2015) and *Cg12* (Svistoonoff et al., 2003) using transgenic plants of *C. glauca* expressing Pro*CgNIN:GFP* and Pro*Cg12:GFP*. *CgNIN* gene is a pre-infection marker which is early expressed in root hairs competent for *Frankia* infection (Clavijo et al., 2015) and *Cg12*, an infection marker associated with root hairs and cortical cells infection by *Frankia* (Svistoonoff et al., 2003).

**Figure 3:**
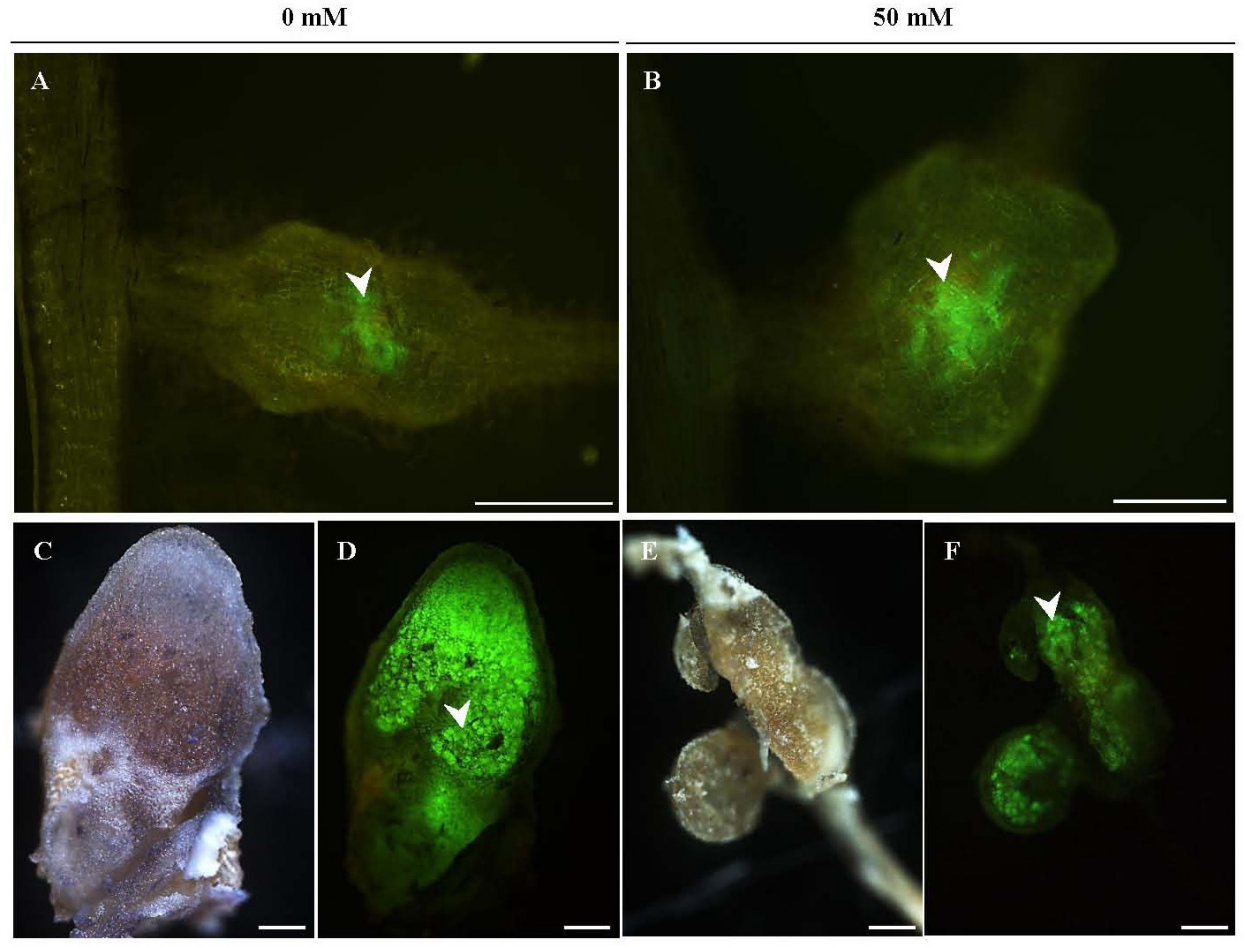
**Pro***Cg12*** is active in saline condition during infection by ***Frankia*****. Salt stress (50 mM NaCl) was first applied then *C. glauca* plants were inoculated separately with *Frankia* strains Ccl3 **(A–D)** and CeD **(E,F)**. Pro*Cg12* is activated in prenodules **(A,B)** and nodules **(C–F)** of control and NaCl treated *C. glauca* plants, 14 days after inoculation. **(C–F)** Sections of matures nodules expressing green fluorescent protein (GFP). White arrows indicate reporter gene expression. **(C,E)** Bright field microscopy. **(A,B,D,F)** Epifluorescence microscopy. Bars 100 μm.

Observations revealed that Pro*CgNIN:GFP* was activated in both control and 50 mM NaCl treated plants, from 24 to 72 h after inoculation with *Frankia* strains CcI3 and CeD (Table 2A), suggesting that *CgNIN* expression was not repressed by salt treatment (50 mM NaCl). Expression of Pro*Cg12:GFP* was observed 14 days after inoculation in both control and 50 mM NaCl treated plants. A lower number of fluorescent spots per plant were detected in NaCl treated plants (Table 2B), pointing to a possible inhibition of infection and Pro*Cg12* expression by salinity. We were not able to detect any differences regarding the pattern or the intensity of Pro*Cg12* activation in prenodules or nodules when comparing control and NaCl-treated plants, as shown in Figure 3.

**Table 2:** **Effect of salt stress on Pro***CgNIN*** and Pro***Cg12*** genes activation**.

**(A)**					
**Transgenic line**	***Frankia* strains**	**NaCl treatments (mM)**	**Hours after inoculation**		
			**24**	**48**	**72**
Pro*CgNIN: GFP*	CcI3	0	+	+	+
		50	+	+	+
	CeD	0	+	+	+
		50	+	+	+
**(B)**					
**Transgenic line**	***Frankia* strains**	**NaCl treatments (mM)**	**Days after inoculation**		
			**3**	**7**	**14**
Pro*Cg12: GFP*	CcI3	0	−	−	+ + +
		50	−	−	+
	CeD	0	−	−	+ + +
		50	−	−	+

*Activation of ProCgNIN:GFP **(A)** and ProCg12:GFP **(B)** genes in presence of 0 and 50 mM of NaCl. Reporter gene expression (GFP) was detected using an epifluorescence microscopy. More sign + indicate more fluorescent spots observed per plant in transgenic lines*.

*+, gene activation; −, inactivation of gene*.

### Nodulated *C. glauca* and *C. equisetifolia* plants are more tolerant to salt stress

**Figure 4:**
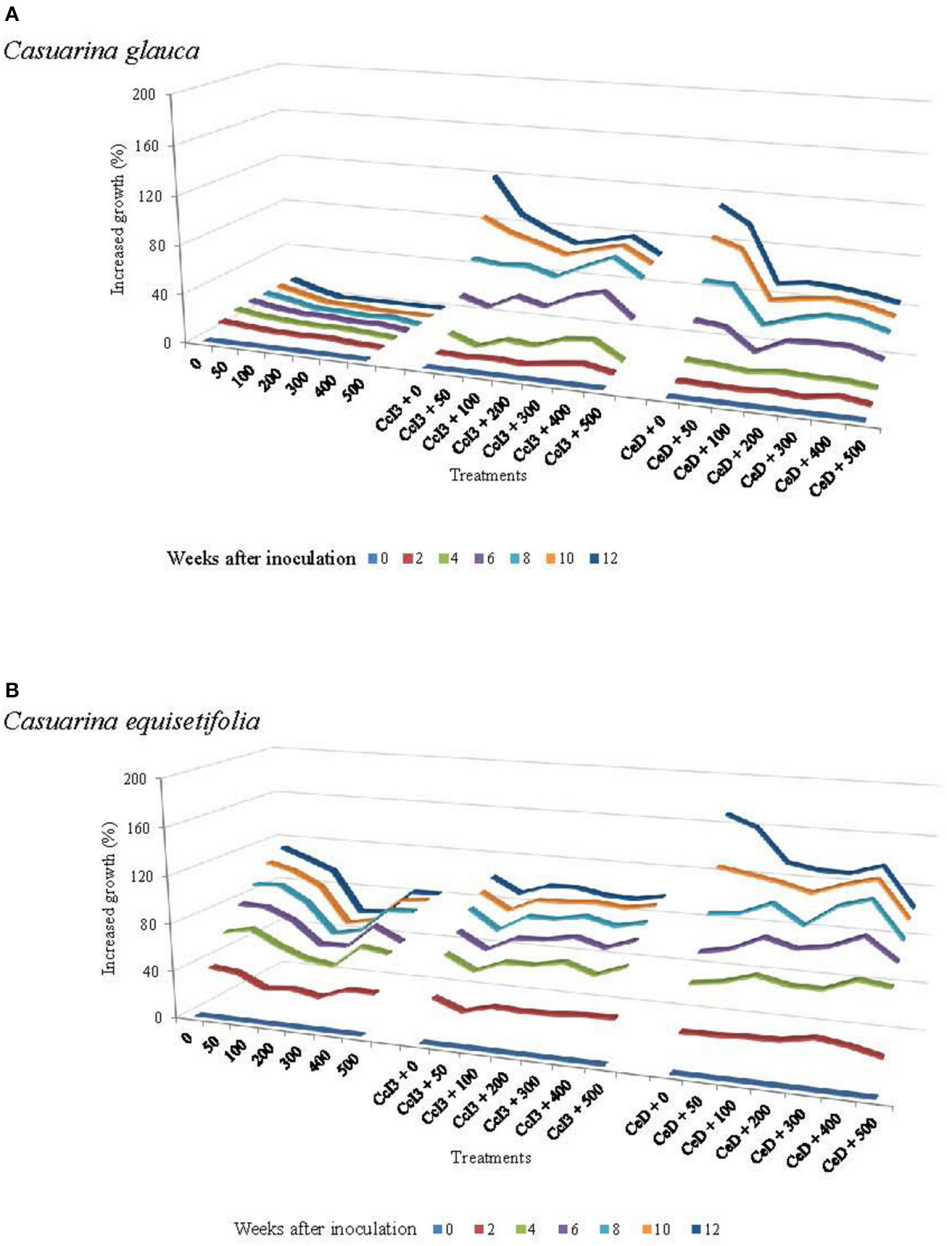
**Shoot growth of non-nodulated and nodulated ***C. glauca*** (A) and ***C. equisetifolia*** (B) plants treated with various salt concentrations**. Plants were inoculated separately with *Frankia* strains Ccl3 and CeD. Salt stress was applied gradually after the establishment of symbiosis. For each treatment, height growth was measured every 2 weeks from the day of inoculation and the increased growth was calculated from this time (0% of growth). Each value represents the mean of plants used in each treatment (*C. glauca n* = 22, *C. equisetifolia n* = 8).

In addition to *C. glauca, C. equisetifolia* was studied because it is the most introduced *Casuarina* species worldwide for land reclamation and reforestation programs including Senegal (LADA, 2003; National Research Council, 1984). Furthermore, *C. equisetifolia* is also highly tolerant to salt stress (El-Lakany and Luard, 1983). The effects of prior inoculation with *Frankia* strains CcI3 and CeD on the salt tolerance of *C. glauca* and *C. equisetifolia* was studied to see if these nitrogen-fixing bacteria could be used to increase the salt tolerance of *Casuarina* species. Both *Frankia* strains CcI3 and CeD improved the growth of *C. glauca* at all concentrations of NaCl tested compared to the control plants (Figure 4A). In control plants, growth decreased with increased NaCl concentrations. There was no growth above 50 mM NaCl, 12 weeks after inoculation. Positive effect of inoculation with *Frankia* CcI3 and CeD on plant height started to be observed 4 weeks after inoculation. Plant height increased progressively at all NaCl concentrations in inoculated plants compared to the control, but growth gradually decreased with increasing NaCl concentrations. For instance, an increase by 65.5 and 44.5% was observed in 500 mM NaCl treated plants inoculated with *Frankia* strains CcI3 and CeD, respectively compared to controls. In contrast, in *C. equisetifolia* plants only *Frankia* strain CeD increased plant growth at all NaCl concentrations as compared to the control (Figure 4B).

Inoculation with *Frankia* strains CcI3 and CeD improved *C. glauca* shoot and total biomass significantly in all NaCl treatments compared to control (Table 3A). Compared to control, *Frankia* strains CcI3 and CeD significantly increased root biomass in all NaCl treatments except for 0, 50, and 100 mM salt-treated plants inoculated with CeD. In both control and inoculated plants, root, shoot, and total biomass decreased with increasing salt concentration, but the change was not significant in plants inoculated with strain CeD and in root dry biomass of control plants. On the other hand, only CeD increased significantly shoot and total dry biomass of *C. equisetifolia* plants for some NaCl treatments (0–200 mM) compared to the control and the plants inoculated with CcI3 (Table 3B). As observed for *C. glauca* plants, root, shoot, and total dry biomass decreased with increasing salt concentration in both control and inoculated plants. The change was significant between NaCl treatments in general and between low (0 and 50 mM NaCl) and high salinity (300 and 500 mM NaCl) in particular.

**Table 3:** **Mean comparison of shoot and root dry weight of non-nodulated and nodulated ***C. glauca*** (A) and ***C. equisetifolia*** (B) plants treated with several salt concentrations**.

**(A)** ***C. glauca***				
	**NaCl treatments (mM)**	**Dry weight (g)**		
		**Control**	**CcI3**	**CeD**
Shoot	0	0.449 c A	1.322 a A	0.876 b A
	50	0.364 c AB	1.103 a AB	0.800 b A
	100	0.320 c B	1.111 a AB	0.694 b A
	200	0.347 c AB	0.924 a B	0.708 b A
	300	0.330 c B	0.920 a B	0.660 b A
	400	0.291 c B	0.921 a B	0.661 b A
	500	0.244 c B	0.836 a B	0.697 b A
Root	0	0.238 b A	0.422 a A	0.272 b A
	50	0.222 b A	0.359 a AB	0.264 b A
	100	0.204 b A	0.354 a AB	0.241 b A
	200	0.192 b A	0.284 a B	0.258 a A
	300	0.190 b A	0.291 a B	0.259 a A
	400	0.177 c A	0.309 a B	0.230 b A
	500	0.174 b A	0.256 a B	0.272 a A
Total biomass	0	0.687 c A	1.744 a A	1.148 b A
	50	0.586 c AB	1.462 a AB	1.064 b A
	100	0.524 c AB	1.465 a AB	0.935 b A
	200	0.539 c AB	1.208 a BC	0.966 b A
	300	0.520 c AB	1.211 a BC	0.919 b A
	400	0.468 c B	1.230 a BC	0.891 b A
	500	0.418 b B	1.092 a C	0.969 a A
**(B)** ***C. equisetifolia***				
Shoot	0	2.813 b A	3.220 b A	5.252 a A
	50	2.336 b AB	2.351 b B	3.432 a B
	100	2.159 b ABC	2.106 b BC	2.804 a BC
	200	1.828 b BC	1.882 b CD	2.518 a CD
	300	1.538 a BC	1.790 a CD	1.918 a D
	400	1.531 a BC	1.496 a D	1.838 a D
	500	1.349 a C	1.384 a D	1.696 a D
Root	0	1.136 a A	1.375 a A	1.249 a A
	50	0.730 b B	1.026 ab B	1.170 a A
	100	0.850 a B	0.778 a C	0.940 a B
	200	0.614 a B	0.689 a CD	0.788 a B
	300	0.553 a B	0.595 a CD	0.544 a C
	400	0.595 a B	0.544 a CD	0.503 a C
	500	0.575 a B	0.395 a D	0.464 a C
Total biomass	0	3.949 b A	4.595 b A	6.501 a A
	50	3.066 b B	3.377 b B	4.602 a B
	100	3.009 b BC	2.884 b C	3.744 a BC
	200	2.442 b BC	2.571 b CD	3.306 a CD
	300	2.091 a BC	2.385 a CD	2.462 a DE
	400	2.126 a BC	2.040 a DE	2.341 a DE
	500	1.924 a C	1.779 a E	2.160 a E

*Each value represents the mean of plants used in each treatment (C. glauca n = 22, C. equisetifolia n = 8). For each salt concentration, different lowercase letters (a–c) indicate significant difference between control and plants inoculated separately with Frankia CcI3 and CeD. For each condition (control/plants inoculated with each strain), different capital letters (A–E) indicate significant difference between NaCl treatments according to the Student-Newman-Keuls (SNK) test at P < 0.05*.

**Figure 5:**
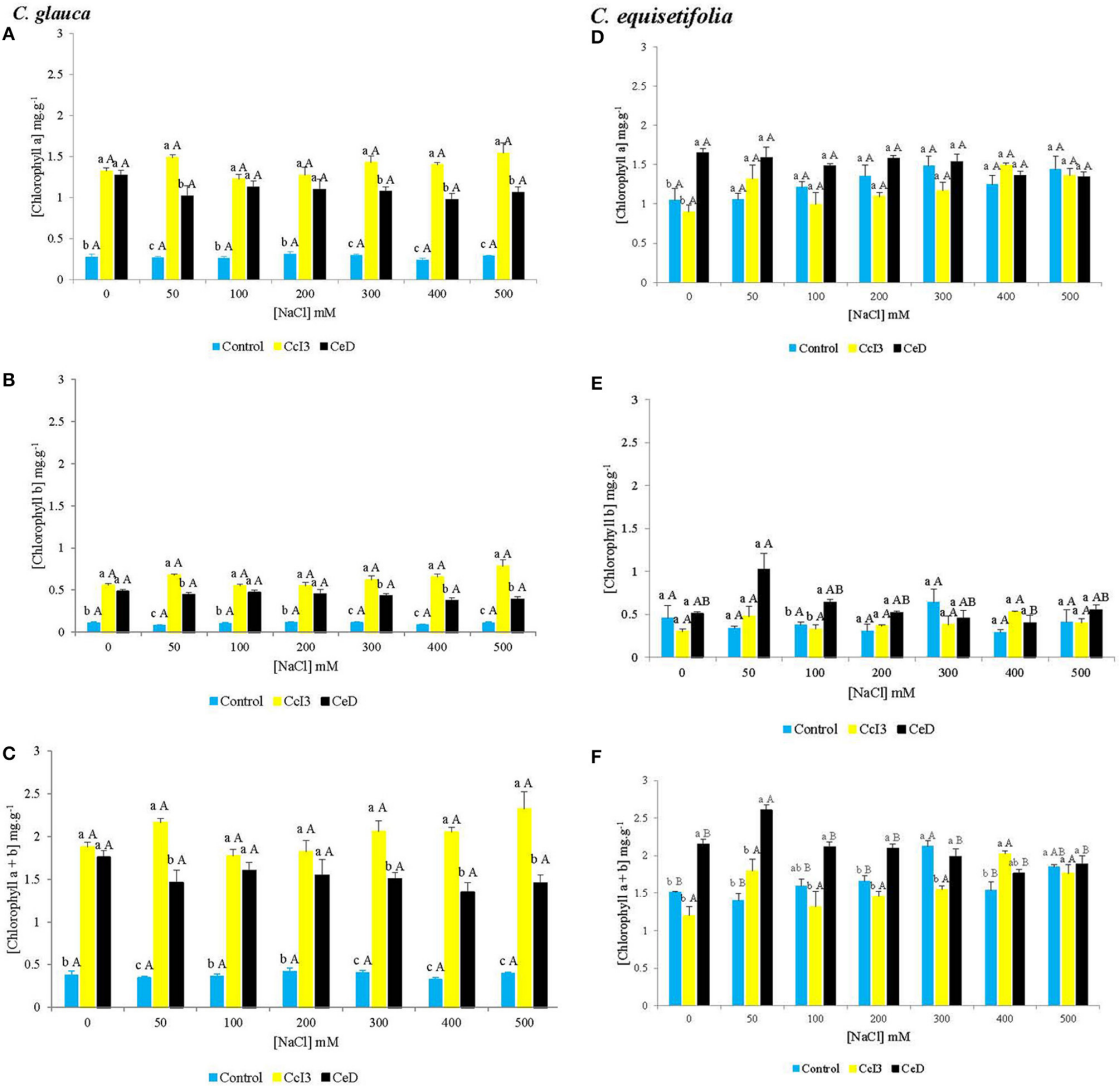
**Mean comparison of chlorophyll contents in non-nodulated and nodulated ***C. glauca*** (A–C) and ***C. equisetifolia*** (D–F) plants under saline conditions**. Each value represents the mean of plants used in each treatment (*C. glauca n* = 5, *C. equisetifolia n* = 4). For each salt concentration, different lowercase letters (a–c) indicate significant difference between control and plants inoculated separately with *Frankia* Ccl3 and CeD. For each condition (control/plants inoculated with each strain), different capital letters (A,B) indicate significant difference between NaCl treatments according to the Student-Newman-Keuls (SNK) test at *P* < 0.05.

Chlorophyll and proline contents were determined in order to appreciate physiological state of non-nodulated and nodulated plants in saline conditions. The chlorophyll content (a, b, and total) was significantly increased in *C. glauca* plants inoculated with *Frankia* strains CcI3 and CeD, as compared to control (Figures 5A–C). However, there was no significant difference between NaCl treatments in *C. glauca* plants. In *C. equisetifolia* plants, the chlorophyll a was significantly increased by strain CeD only in no salt-treated plants (Figures 5D–F). No significant difference was observed between NaCl treatments. However, *Frankia* strain CeD increased the total chlorophyll content in 0, 50, 100, and 200 mM NaCl treated plants, compared to control and plants inoculated with CcI3.

**Figure 6:**
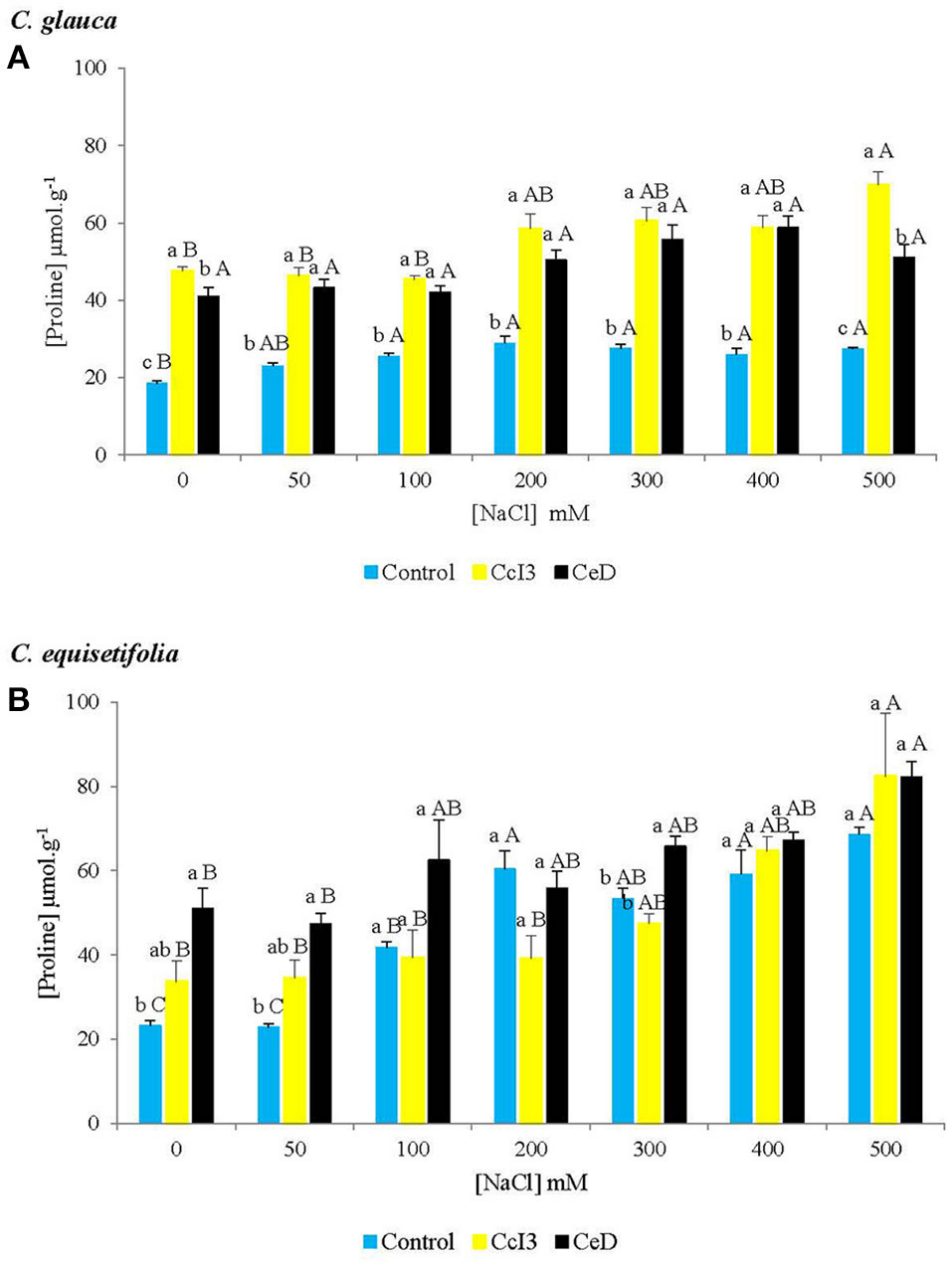
**Mean comparison of proline contents in non-nodulated and nodulated ***C. glauca*** (A) and ***C. equisetifolia*** (B) plants treated with several salt concentrations**. Each value represents the mean of plants used in each treatment (*C. glauca n* = 5, *C. equisetifolia n* = 4). For each salt concentration, different lowercase letters (a–c) indicate significant difference between control and plants inoculated separately with *Frankia* Ccl3 and CeD. For each condition (control/plants inoculated with each strain), different capital letters (A,B) indicate significant difference between NaCl treatments according to the Student-Newman-Keuls (SNK) test at *P* < 0.05.

As what was observed in chlorophyll content, there were significant changes in proline content between control and inoculated plants (Figure 6). *Frankia* strains CcI3 and CeD increased the proline content of *C. glauca* at all concentrations of salt tested (Figure 6A). Proline content increased with increasing salt concentrations in the control and inoculated plants, but the change was not significant in *C. glauca* plants inoculated with strain CeD. Similarly, in *C. equisetifolia* plants, proline content increased with increasing salinity (Figure 6B). There were significant differences between NaCl treatments in both control and plants inoculated with strains CcI3 and CeD. Only *Frankia* strain CeD increased proline contents significantly in 0, 50, and 300 mM NaCl treated plants.

## Discussion

Among *Casuarina* tree species, *C. glauca* and *C. equisetifolia* have been shown to be highly salt-tolerant (Hyland, 1983; Luard and El-Lakany, 1984; Aswathappa and Bachelard, 1986; Van der Moezel et al., 1989) and are widely planted outside of their native habitat (National Research Council, 1984). However, salinity could affect plant growth and the establishment of actinorhizal symbiosis which could be thus a limit to salinized land reclamation (Reddell et al., 1986). In this study, we first investigated the effect of salinity on the symbiotic relationship between *C. glauca* and two contrasting *Frankia* strains CcI3 (salt sensitive) and CeD (salt tolerant).

Our results indicate that nodule formation in *C. glauca* is inhibited by salt stress regardless of the salt tolerance of the *Frankia* strain. However, the most salt tolerant strain, CeD, is still able to infect *C. glauca* up to 200 mM NaCl while the salt-sensitive strain CcI3 is not. Nitrogen fixation was not measured in this study, although it has been reported a significant correlation between nodule number per plant in *C. glauca* and the acetylene reduction activity (ARA) under salt stress (Girgis et al., 1992). A decrease in nodulation under saline conditions has been previously reported for *C. equisetifolia* (Ng, 1987; Tani and Sasakawa, 2003) and *C. obesa* (Reddell et al., 1986) depending on the *Frankia* source, culture conditions and duration of the experiment. Nodulation did not occur in *C. equisetifolia* inoculated with Ceq1 strain and cultured in 500 mM NaCl for 6 weeks (Tani and Sasakawa, 2003), while nodules were formed in C. *equisetifolia* seedlings cultured for 24 weeks at 500 mM NaCl and inoculated with a nodule suspension (Ng, 1987). With *C. obesa*, increased salinity reduced nodule dry weight with both *Casuarina–Frankia* associations having a more pronounced effect with one of the inoculum source (Reddell et al., 1986).

The effects of salinity on *C. glauca* nodulation could be due to an inhibition of nodule initiation and/or infection processes. These processes leading to the development of root nodules of *Casuarina* involve various responses such as root hair deformation (Torrey, 1976) and early expression of several genes like *CgNIN* and *Cg12* (Laplaze et al., 2000; Svistoonoff et al., 2003; Clavijo et al., 2015). Extensive deformation of root hairs occurs in the zone of root hair elongation within the first 24 h after inoculation (Torrey, 1976). *Frankia* hyphae infect plants through the intracellular infection pathway in *Casuarina* trees (Callaham et al., 1979; Perrine-Walker et al., 2011). We observed that an increase in salt concentration reduced the percentage of root hairs deformed in *C. glauca* plants, 48 h after inoculation with both strains and with a more pronounced effect in plants inoculated with the salt-sensitive strain CcI3. Root hair deformation is dependent on the production of diffusible signals by *Frankia* (Cérémonie et al., 1999). The observed results might be due to the fact that the salt tolerant strain CeD is able to maintain growth and production of symbiotic factors at higher salt concentration than CcI3. This effect could explain in part the impact of salt on nodule formation. Indeed, there is a positive correlation between the extent of root hair deformation and the number of nodules which subsequently developed (Callaham et al., 1979). In addition, salt stress decreased the number and size of root hairs regardless of the presence of *Frankia*, which could reduce their availability and susceptibility. Therefore, *Frankia* colonization may decrease and the establishment of the symbiosis is thus impaired. A similar reduction in root hair deformation by salt stress was reported in legumes *Vicia faba* (Zahran and Sprent, 1986), *Glycine max* (Tu, 1981), and *Medicago sativa* (Lakshmi-Kumari et al., 1974) in response to rhizobial inoculation. The extent of the deformation depends on the association *Rhizobium*-Legume and was correlated to the number or dry weight of nodules. Morphological symptoms of damage by NaCl such as reduction in the number and size of root hairs was observed in *Medicago sativa* (Lakshmi-Kumari et al., 1974).

*CgNIN* is a transcription factor which plays a central role in the nodulation of actinorhizal hosts and is induced by diffusible symbiotic signals produced by *Frankia* (Clavijo et al., 2015; Chabaud et al., 2016). *Cg12* is a subtilisin gene isolated from *C. glauca* and its expression is associated with *Frankia* infection (Laplaze et al., 2000; Svistoonoff et al., 2003). In this study, we showed that *CgNIN* was activated in both control and 50 mM NaCl treated plants (Supplementary Figure 1), from 24 to 72 h after inoculation. This effect suggests that the production of symbiotic diffusible signals by *Frankia* or its perception is not perturbed by mild salt stress for both strains. On the other hand, expression of *Cg12* was observed 14 days after inoculation in both treatments with low number of fluorescent spots in NaCl treated plants. This effect suggests *Cg12* expression is negatively affected by salinity that is possibly related to a perturbation of plant cell infection. This result is in accordance with those of Duro et al. (2016) which showed that *Cg12* was down-regulated with increasing salt concentration. However, this study used higher levels of NaCl (200, 400, and 600 mM) that what we used in this experiment (50 mM).

Altogether, our results indicate that salt stress alters actinorhizal symbiosis formation in *C. glauca*. This effect could be due at least in part to a negative impact of salt stress on the infection process that might be related to a reduction of potential infection sites (root hairs) or reduced perception of infection signals. Furthermore, the salt-tolerant strain CeD is able to infect at higher concentrations of salt than the salt sensitive strain CcI3. This result indicates that the use of appropriate strains is necessary for efficient nodulation of trees in salinized soils.

The impact of *Frankia* inoculation on salt tolerance in *C. glauca* and *C. equisetifolia* was tested. Our results indicate that inoculation of *C. glauca* and *C. equisetifolia* by *Frankia* strains CcI3 and CeD significantly improved plants growth under salt stress, depending on the specific *Casuarina*-*Frankia* association. For *C. glauca*, both *Frankia* strains significantly increased plant height, shoot, root and total dry weight at all concentrations of NaCl, as compared to uninoculated plants. This positive effect was more pronounced in plants inoculated with strain CcI3. In contrast, only *Frankia* strain CeD increased *C. equisetifolia* height at all NaCl treatments, and significantly elevated plant shoot, root, and total dry weight from 0 to 200 mM NaCl, as compared to control. These results suggest that the effectiveness of the symbiosis in saline conditions depends on the appropriate *Casuarina-Frankia* association. Indeed, according to Girgis et al. (1992), there is no correlation between *in vitro* salt tolerance of *Frankia* strains and their effectiveness in association with plants under salt-stressed conditions. However, it is important to emphasize that the experiment with *C. glauca* was conducted in hydroponic conditions, whereas *C. equisetifolia* was grown in soil. The improvement of morphological parameters (height, shoot, root and total dry weight) may be due to the increased N nutrition and photosynthesis potential in *Casuarina* inoculated with *Frankia* compared to the uninoculated controls. This conclusion was supported by our results for chlorophyll (a, b, and a + b) content and nitrogenase activity under saline conditions. Under all NaCl concentrations, chlorophyll content was significantly increased in *C. glauca* plants inoculated with both strains, as compared to control. With *C. equisetifolia*, only CeD increased significantly total chlorophyll content from 0 to 200 mM NaCl. Salinity decreased nitrogenase activity in *C. glauca* (Supplementary Figure 2). However, N_2_ fixation occurred even at the highest NaCl concentration (Supplementary Figure 2). This implies that increased N nutrition and potential photosynthesis allow inoculated *Casuarina* plants to grow better than uninoculated controls plants under saline conditions. These results are in agreement with a previous report showing that the actinorhizal tree *Alnus glutinosa* inoculated with *Frankia* and cultivated in alkaline and saline anthropogenic sediment, had better plant growth, leaf N and chlorophyll a + b content than the control (Oliveira et al., 2005). Several studies have shown that inoculation with selected microsymbionts like *Frankia* can enhance the development of actinorhizal plants and their resistance to other abiotic stresses such as heavy metals and extreme pH and temperature (Reviewed by Ngom et al., 2016). Symbiotic associations with arbuscular mycorrhizal fungi (AMF) and nitrogen-fixing bacteria called rhizobia can also enhance plant salinity tolerance, leading to better plant growth and yield, nutrient acquisition and chlorophyll content in several species including *Medicago sativa* (Azcon and El-Atrash, 1997), *Acacia nilotica, Leucaena leucocephela, Prosopis juliphora* (Bala et al., 1990), *Phaseolus vulgaris* (Dardanelli et al., 2008), and soybean (Elsheikh and Wood, 1995), under saline conditions. The benefits of these microsymbionts in saline environments depend also on the symbiotic associations.

Compatibles solutes or osmolytes such as glycine betaine, mannitol, or proline are accumulated in organisms in response to salt and osmotic stresses (Delauney and Verma, 1993; Wang et al., 2003). They play important roles in maintaining cell turgor and thus the driving gradient for water (Wang et al., 2003). Compatible solutes can also act as free-radical scavengers or chemical chaperones by directly stabilizing membranes and/or proteins (Lee et al., 1997; Bohnert and Shen, 1998; McNeil et al., 1999; Diamant et al., 2001). Proline, an amino acid, is the most common osmolyte accumulated under salinity and drought stress in plants (Watanabe et al., 2000; Tani and Sasakawa, 2003). In our study, a significantly higher proline content was observed in all inoculated *C. glauca* plants at all NaCl concentrations, as compared to the control. Significant improvement of proline content was also observed in 0, 50, and 300 NaCl treated *C. equisetifolia* plants inoculated with strain CeD. In both control and inoculated plants, proline content increased with increasing salinity. These results suggest that, in addition to better N nutrition and potential photosynthesis, proline accumulation adjusts the osmotic pressure and maintain cell homeostasis in inoculated *C. glauca* and *C. equisetifolia* plants, under saline conditions. These results are in agreement with those of Diouf et al. (2005) which showed that inoculation with both *Rhizobium* and AMF induced higher proline content in legumes such as *Acacia auriculiformis* and *Acacia mangium*, compared to uninoculated plants, at all levels of salinity tested (0, 50, and 100 mM NaCl). Proline accumulation under salt stress has been previously described in *C. equisetifolia* seedlings not infected by *Frankia* (Tani and Sasakawa, 2006).

In conclusion, our results strongly indicate that the beneficial effects of *Frankia* inoculation are due to improved N nutrition, photosynthesis potential and proline accumulation in inoculated plants under salt stress conditions. There was no correlation between *in vitro* salt tolerance of *Frankia* strains and efficiency *in planta* in salt stress conditions. Hence, the success of planting *Casuarina* in saline sites will require appropriate salt-tolerant *Casuarina-Frankia* associations that will form an efficient N_2_-fixing symbiosis. *In vitro* salt tolerance of *Frankia* strains should be considered if they are introduced in saline soils, otherwise, the screening should be done with both symbiotic partners.

## Author contributions

MN conducted some experiments, analyzed the data, interpreted the results, and prepared the manuscript. KG and JF conducted some experiments and prepared the manuscript. ND, HG, VH, SS conducted some experiments, interpreted the results, and improved the manuscript. RO, LL, and LT interpreted the results and improved the manuscript. AC and MS designed and coordinated the experiments, analyzed the data, interpreted the results, and improved the manuscript.

### Conflict of interest statement

The authors declare that the research was conducted in the absence of any commercial or financial relationships that could be construed as a potential conflict of interest.
