# Supplementary material for: Symbiotic Performance of Diverse Frankia Strains on Salt-Stressed Casuarina glauca and Casuarina equisetifolia Plants
Source: Front Plant Sci. 2016 Aug 31;7:1331. doi: 10.3389/fpls.2016.01331 (PMC5006599; doi:10.3389/fpls.2016.01331)
Supplement: Supplementary file 1 [file Image1.PDF]

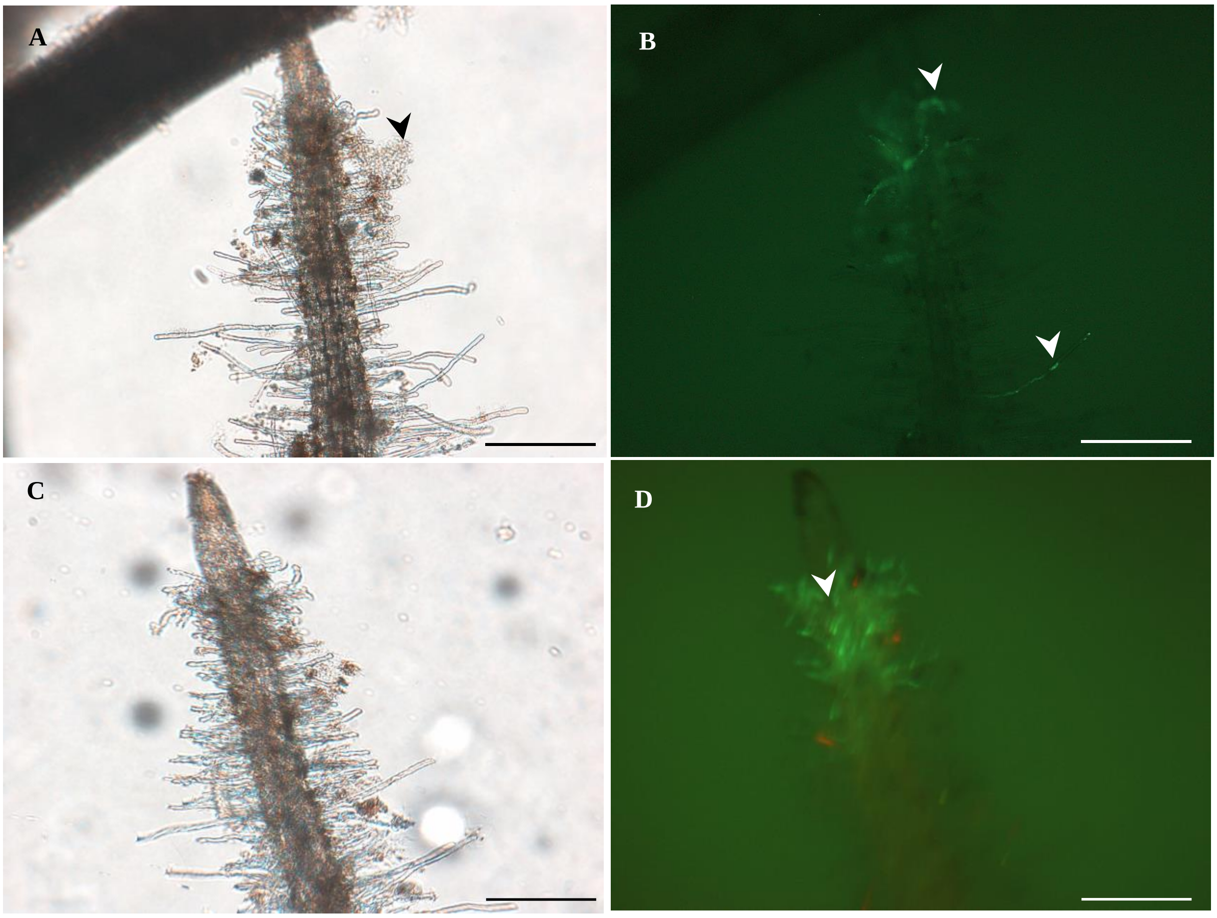

Figure 1 | Pro*CgNIN* is active in saline condition during infection by *Frankia*. Salt stress (50 mM NaCl) was first applied then *C. glauca* plants were inoculated separately with *Frankia* strains CcI3 or CeD. Pro*CgNIN* is activated in root hairs of control **(B)** and NaCl-treated *C. glauca* plants **(D)** from 24 hours after inoculation. White arrows indicate reporter gene expression, black arrow indicate *Frankia*. **(A, C)** Bright field microscopy. **(B, D)** Epifluorescence microscopy. Bars, 100 µm.

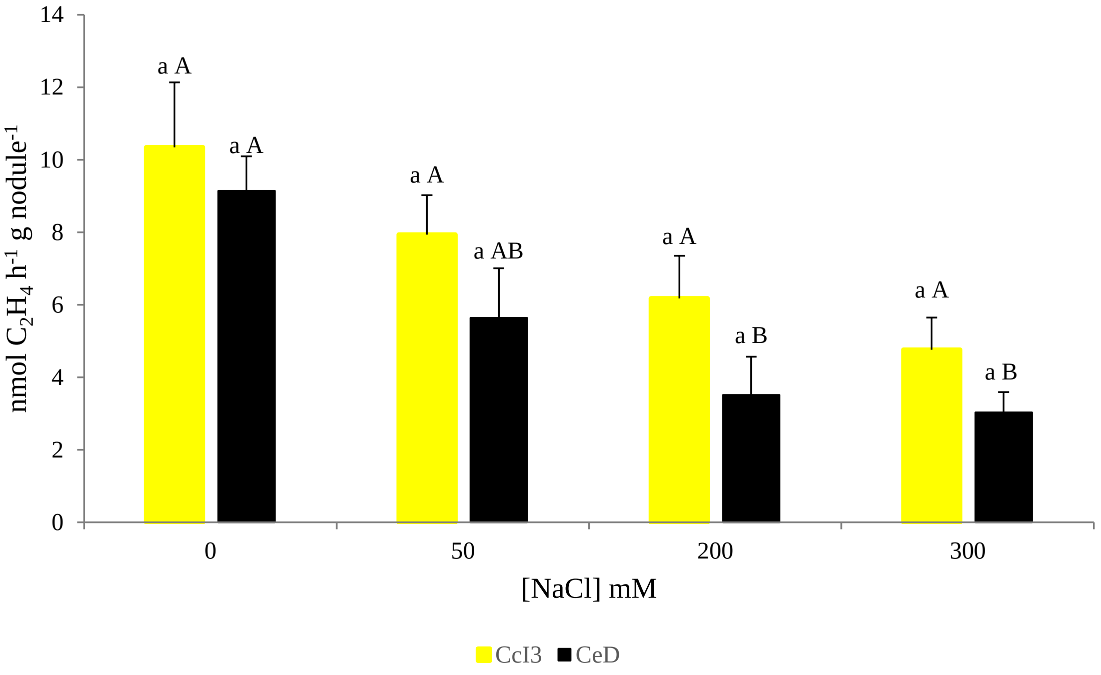

Figure 2 | Acetylene Reduction Assay on *C. glauca* plants inoculated with *Frankia* strains CcI3 and CeD. Each value represents the mean of plants used in each treatment (n = 5). For each salt concentration, different lowercase letters (a, b) indicate significant difference between plants inoculated separately with *Frankia* CcI3 and CeD. For each condition (plants inoculated with each strain), different capital letters (A, B) indicate significant difference between NaCl treatments according to the Student-Newman-Keuls (SNK) test at $P < 0.05$.
